# Novel Reassortant Avian Influenza A(H9N2) Virus Isolate in Migratory Waterfowl in Hubei Province, China

**DOI:** 10.3389/fmicb.2020.00220

**Published:** 2020-02-13

**Authors:** Xiang Li, Jing Sun, Xinru Lv, Yajun Wang, Yulei Li, Minghui Li, Wei Liu, Min Zhi, Xiaoyu Yang, Tian Fu, Peiran Ma, Yi Li, Xihua Zhou, Yong Li, Guoxiang Yang, Guang Chen, Jun Zhang, Hesong Zheng, Guogang Zhang, Yuping Hua, Siyuan Yang, Yanbing Li, Juergen A. Richt, Hongliang Chai

**Affiliations:** ^1^College of Wildlife and Protected Area, Northeast Forestry University, Harbin, China; ^2^State Key Laboratory of Veterinary Biotechnology, Harbin Veterinary Research Institute of Chinese Academy of Agricultural Sciences, Harbin, China; ^3^Investigation and Planning Institute of Hubei Forestry, Wuhan, China; ^4^Hubei Wildlife Rescue, Research and Development Center, Wuhan, China; ^5^Research Institute of Forest Ecology, Environment and Protection, Chinese Academy of Forestry, Beijing, China; ^6^Heilongjiang Vocational College for Nationalities, Harbin, China; ^7^Diagnostic Medicine/Pathobiology, Center of Excellence for Emerging and Zoonotic Animal Diseases (CEEZAD), College of Veterinary Medicine, Kansas State University, Manhattan, KS, United States

**Keywords:** H9N2 subtype, influenza in birds, phylogeny, public health, reassortant viruses

## Abstract

In December 2017, an influenza A(H9N2) virus (B51) was isolated from migratory waterfowl in Hubei Province, China. Phylogenetic analysis demonstrated that B51 is a novel reassortant influenza virus containing segments from human H7N4 virus and North American wild bird influenza viruses. This suggest that B51 has undergone multiple reassortment events.

## Introduction

The H9N2 influenza virus was initially isolated from turkeys in Wisconsin in 1966 (Homme and Easterday, 1970). Since then, H9N2 subtype avian influenza viruses (AIVs) have spread worldwide and have become endemic in poultry in Europe, Asia and Africa (Li et al., 2017). H9N2 subtype AIVs can infect wild and domestic birds and cross the species barrier to mammals, including humans (Li et al., 2014). In particular, reassortant AIVs with partial or whole H9N2 internal gene sequences can spread across various host species (Li et al., 2014). During the global highly pathogenic avian influenza (HPAI) H5N1 epidemic that started in Hong Kong in 1997, and during the five waves of the H7N9 outbreaks since 2013, it was determinated that H9N2 viruses were found to have donated whole internal genes to the H5N1 and H7N9 viruses (Guan et al., 1999; Lam et al., 2013). The H9N2 virus was also reported to provide internal gene segments to the fatal human H10N8 (Chen et al., 2014) and H5N6 (Shen et al., 2016) reassortants, thus posing a substantial threat to public health.

In 2005, the first large-scale wild bird die-off due to H5N1 highly pathogenic avian influenza viruses (HPAIVs) infection occurred at Qinghai Lake, this caused widespread public concern about the spread of HPAIVs by wild birds (Liu et al., 2005). As wild birds are the natural host and the reservoir of AIVs, their migration is the driving force of AIV distribution and transmission. A subtype H5N8 virus was detected in poultry in South Korea in 2013/2014, and rapidly spread worldwide in 2014/2015, even to the United States. In addition, interdisciplinary analysis showed that long-distance migration of wild birds can play a major role in the global spread of highly pathogenic clade 2.3.4.4 H5N8 (Global Consortium for H5N8, and Related Influenza Viruses, 2016). Three AIV intercontinental distribution events (H9N2, H8N4, H5N8) suggest that viral spread by migratory birds crossing the Bering Strait might be common (Ramey et al., 2018). However, the significance of wild birds carrying and spreading AIVs across continents to public health is still unclear.

## Materials and Methods

### Sampling, Virus Isolation and Sequencing

Hubei Province, located in central China, has widely distributed lakes and wetlands providing a favorite habitat for migratory birds on the East Asia-Australasian flyway (Supplementary Figure S3). In the winter and spring, migratory waterfowl (mainly Anseriformes, Ciconiiformes, and Podicipediformes) aggregate at lakes or wetlands, making it an ideal sampling site for AIVs. Fresh droppings, oropharyngeal and cloacal swabs of waterfowl were sampling.

The samples were oscillated and then centrifuged, and the collected supernatant was inoculated into 9-day-old specific pathogen-free (SPF) chicken embryos. Then, 72 h after incubation, the allantoic fluid was harvested, and the hemagglutinin (HA) activity was assayed. Viral RNA was extracted from HA positive samples from incubated allantoic fluid using a QIAamp Viral RNA Mini Kit (Qiagen, Germany), reverse transcribed using the primer Un12 and subjected to RT-PCR using the method described in the WHO manual (World Health Organization [WHO], 2002) to confirm AIV positive. The PCR products of eight fragments of the isolates were sequenced using a set of specific sequencing primers listed in a previous dissertation (Chai, 2012). The sequence data were compiled using the SeqMan program (DNASTAR, Madison, WI, United States).

### Genetic Analysis

A BLASTn search was performed against sequences in the GISAID database to identify the closest relatives of the H9N2 isolate on October 23, 2018, and eight datasets were derived from the top 100 BLASTn hits. Sequences were aligned using MUSCLE with manual adjustments (Edgar, 2004). The alignment lengths for each dataset were: PB2 2,280 nucleotides (nt), PB1 2,274 nt, PA 2,151 nt, HA 1,683 nt, NP 1,497 nt, NA 1,410 nt, M 982 nt, NS 838 nt. For all eight datasets, sequences without full alignment length were removed. A phylogenetic tree was reconstructed by maximum likelihood (ML) method in RAxML under the GTRGAMMA model with 1,000 bootstrap replicates (Stamatakis, 2014). The time of the most common ancestor was estimated with BEAST package (v1.8.4) (Drummond and Rambaut, 2007), with the SRD06 nucleotide substitution model (Shapiro et al., 2006), an uncorrelated lognormal relaxed clock model (Drummond et al., 2006), a Constant Size coalescent model (Kingman, 1982). A Markov chain Monte Carlo (MCMC) run of 50,000,000 states sampling each 5,000 steps were combined to obtain an effective sample size of ≥200. Maximum clade credibility (MCC) trees were reconstructed with 10% burn-in and annotated with FigTree (v1.7.1).

### Receptor Binding Specificity Assays

The receptor binding specificity of the B51 virus was determined via HA assays involving 1% chicken red blood cells (cRBCs) and sheep red blood cells (sRBCs). The cRBCs surface contains α-2, 3-linked and α-2, 6-linked sialic acid receptors. While, the sheep red blood cells surface only contains α-2,3-linked sialic acid receptors. For HA assays, α-2, 3-linked sialic acid receptors on cRBCs were enzymatically removed using an α-2, 3-specific sialidase, and only α-2, 6-linked human sialic acid receptors were retained. Other cRBCs were treated with VCNA to eliminate both α-2, 3-linked and α-2, 6-linked sialic acid receptors. Viruses were serially diluted in 50 μL PBS and mixed with 50 μL of 1% red blood cells in a 96-well plate. HA titers were measured after a 20-min reaction at room temperature1.

### Mouse Studies

Six-week-old female BALB/c mice were obtained from Beijing Vital River Laboratories. For the 50% minimum lethal dose (MLD_50_) testing of B51, groups of five BALB/c mice were anesthetized with CO_2_ and inoculated intranasally with 10-fold serial dilutions containing 10^6^ to 10^1^ 50% egg infectious doses (EID_50_) of B51 in a volume of 50 μl. The mice were monitored for 14 days to assess mortality. To detect the infectivity of B51 in mice, three mice were anesthetized with CO_2_ and instilled intranasally with 10^6^ EID_50_ of the virus in a volume of 50 μl. Three mice were euthanized at 3 days post infection (dpi), and different tissues, including the brain, spleen, kidney, lung (right lungs) and nasal turbinate, were collected. The tissue samples were homogenized and centrifuged at 8,000 rpm. Then, the supernatants were collected and inoculated into three 9-day-old eggs. 72 h after incubation at 37°C, the activity of HA was tested, and the EID_50_ was determined using the Reed-Muench method. A/chicken/Heilongjiang/A147/2016(H9N2) was set as the positive control.

## Results

From December 2017 to March 2018, a total of 2298 samples, including 1905 fresh droppings and swabs, were collected from migratory waterfowl in an active AIV surveillance program in Hubei Province, China. 7 samples were positive for AIV according to RT-PCR detections, following combined subtypes: H1N3, H4N6, and H9N2, and the total AIV positivity rate was 0.305% (7 of 2298, Supplementary Table S1). All AIVs were recovered from fresh droppings. We selected the single H9N2 isolate, A/wild waterfowl/Hubei/B51/2017(H9N2) (B51), for genomic characterization as part of this investigation. The whole-genome sequences were submitted to GISAID EpiFlu database (accession numbers: EPI1364378-EPI1364385).

The amino acid sequence PAASDR↓GLFGAI was found within the cleavage site of the HA protein of B51, which is a characteristic of low pathogenic AIV in chicken. The amino acid substitutions Q226L and G228S (H3 numbering) were not found, suggesting that they prefer avian-like receptors (Vines et al., 1998). Residues Q591, E627, and D701 in the PB2 protein suggest that these viruses have not yet adapted to mammalian hosts (Subbarao et al., 1993; Li et al., 2005; Yamada et al., 2010). Furthermore, pathogenicity associated mutations (N30D, T215A in M1; P42S, L98F, I101M in NS1) were observed, which might enhance virus virulence in mice (Jiao et al., 2008; Fan et al., 2009; Kuo and Krug, 2009).

To determine the origin of the H9N2 B51 isolate, we sequenced the entire genome and constructed eight individual maximum-likelihood phylogenetic trees (Supplementary Figure S1). The PB1, HA, and NA gene segments were closely related to North American H10Nx, H9N2, and H3N2 viruses, respectively, isolated from wild birds (Supplementary Figures S1B,D,F). The HA and NA genes of B51 were clustered with influenza A viruses dispersed across a relatively large geographic region (East Asia and Northern America) over a long period circulating in wild waterfowl. The PB2 gene was closely related to a Chinese wild bird H6N8 virus, and the PA and M genes were closely related to poultry and human H7N4 viruses (Supplementary Figures S1A,C,G). The NP and NS genes originated from H6N6/H4N6 and H11N3 subtype influenza viruses, respectively, isolated from ducks in Japan (Supplementary Figures S1E,H). This demonstrates that the B51 H9N2 virus is a reassortant virus carrying gene segments from various influenza A virus subtypes and various continents (Asia and North America).

**FIGURE 1:**
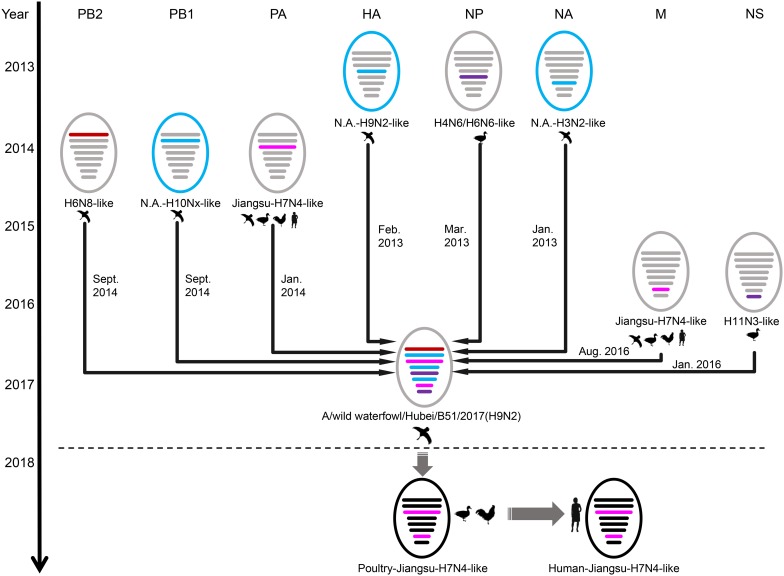
Schematic representation of the H9N2 reassortant virus detected in Hubei Province, China, and its relation to human H7N4 virus and North America viruses. The eight gene segments were (horizontal bars starting from top to bottom of the “virion”) polymerase basic 2, polymerase basic 1, polymerase acidic, hemagglutinin, nucleoprotein, neuraminidase, matrix, and non-structural. North America (N.A.)-origin segments are indicated with blue bar, Japan-origin segments are indicated with purple bar, Jiangxi-origin segment is indicated with red bar, and H7N4 related segments are indicated with pink bar. Circles outside are colored according to their location, viruses isolated from Eurasia are colored in gray or black, and viruses isolated from North America are colored in blue.

BEAST software was used to determine the time of the most recent common ancestor (tMRCA). As shown in Supplementary Table S2 and Supplementary Figure S2, reassortment events to produce the novel H9N2 virus happened between 2013 and 2016. Interestingly, the HA, NP, and NA segments were obtained in January-March 2013 (Table 1 and Supplementary Figures S2D–F), whereas the PB2, PB1, and PA segments emerged in January–September 2014 (Table 1 and Supplementary Figures S2A–C). The M segment was obtained from a duck H5N3 virus (Li et al., 2019) in August 2016 and the NS segment was obtained from a duck H11N3 virus in early 2016 (Table 1 and Supplementary Figures S2G,H). In addition, we analyzed the Jiangsu poultry and human H7N4 viruses using BEAST software to determine the tMRCA, and found that the PA and M segments of the human H7N4 virus were most likely obtained in January/March 2017, respectively (Table 1 and Supplementary Figures S2C,G). This indicates that the PA and M genes of the migratory bird H9N2 virus possibly circulated in the Jiangsu poultry AIV gene pool at the beginning of 2017, eventually causing the first case of human infection with influenza A subtype H7N4 virus in December 2017. This indicated that the novel waterfowl-origin H9N2 virus is a reassortant influenza virus containing segments from different geographic regions (Asia, North America), a multitude of subtypes and different host species (various wild waterfowl, domestic poultry) (Figure 1).

**TABLE 1:** Evolution rate and time of most recent common ancestor for each of the eight segments.

**Segment^†^**	**Mean evolution rate (substitution/site/year)**	**95% HPD interval^‡^**	**Most recent common ancestor**	**tMRCA^§^**	**95% HPD interval**	**Posterior probability**
PB2	2.9729E-3	[2.3548E-3, 3.5933E-3]	B51 and Jiangxi wild bird H6N8 strain	September 2014	[February 2013, October 2015]	0.9989
PB1	2.6993E-3	[2.2141E-3, 3.1885E-3]	B51 and New Jersey shorebird H10 strains	September 2014	[September 2013, July 2015]	1
PA	1.8974E-3	[1.5617E-3, 2.2222E-3]	B51, Mongolia duck H3N8 and Jiangsu H7N4 strains	January 2014	[November 2012, February 2015]	1
			Jiangsu H7N4 strains	January 2017	[April 2016, August 2017]	1
HA	2.8731E-3	[2.3557E-3, 3.4235E-3]	B51 and Alaska wild waterfowl H9N2 strains	February 2013	[March 2012, June 2014]	1
NP	1.3318E-3	[1.0718E-3, 1.5977E-3]	B51 and Japan duck strains	March 2013	[June 2012, November 2013]	0.2861
NA	1.841E-3	[1.5529E-3, 2.163E-3]	B51 and Wisconsin wild duck H3N2 strain	January 2013	[January 2012, September 2013]	1
M	1.7085E-3	[1.2008E-3, 2.162E-3]	B51 and Chongqing duck H5N3	August 2016	[December 2015, May 2017]	0.4195
			Jiangsu H7N4 strains	March 2017	[August 2016, August 2017]	0.9948
NS	1.8389E-3	[1.2526E-3, 2.5096E-3]	B51 and Japan duck H11N3 strains	January 2016	[September 2015, June 2016]	0.3531

*^†^PB2, basic polymerase 2; PB1, basic polymerase 1; PA, acidic polymerase; HA, hemagglutinin; NP, nucleoprotein; NA, neuraminidase; M, matrix protein; NS, non-structural protein. ^‡^HPD, highest posterior density. ^§^tMRCA, time of most recent common ancestor.*

**FIGURE 2:**
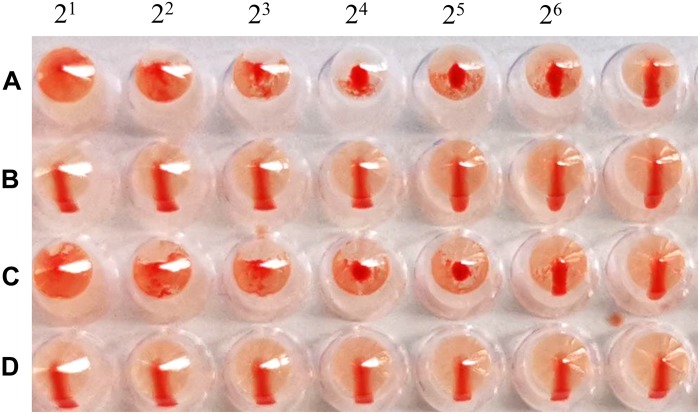
Agglutination of various red blood cell samples by the B51 virus. **(A)** Chicken red blood cells (with α-2, 3-linked sialic acid receptors and α-2, 6-linked sialic acid receptors). **(B)** Chicken red blood cells treated with α-2, 3-sialidase (with only α-2, 6-linked sialic acid receptors). **(C)** Sheep red blood cells (with only α-2, 3-linked sialic acid receptors). **(D)** Chicken red blood cells treated with VCNA (no receptors).

**FIGURE 3:**
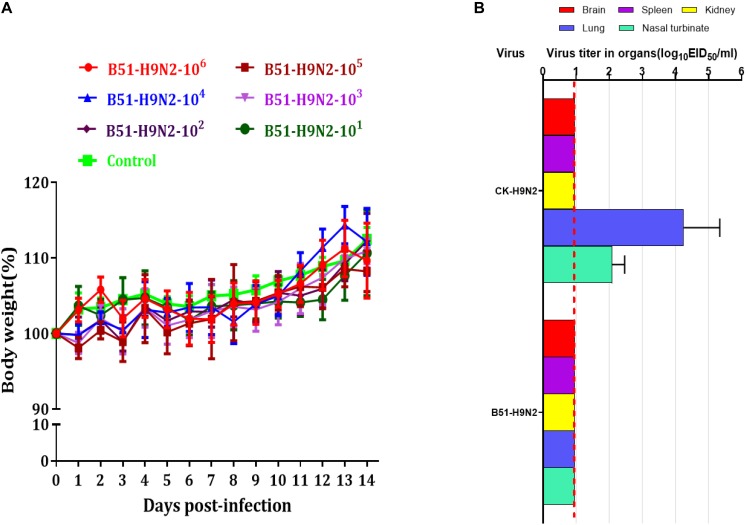
Pathogenicity of B51 virus in mice. **(A)** Body weights were monitored daily for 14 days. Values represent means ± standard deviation (SD) for overall body weight loss compared with initial body weight. **(B)** Viral titers in the various tissues of the mice at 3 dpi. Viral titers are shown as the mean ± standard deviation. The dashed line indicates the lower limit of viral detection.

The cRBCs exhibit α-2,3-linked and α-2,6-linked sialic acid receptors. In contrast, the sheep red blood cells exhibited only α-2,3-linked sialic acid receptors. The results showed that the B51 strain agglutinated chicken and sheep red blood cells but could not agglutinate cRBCs treated with α-2,3-sialidase that have only α-2,6-linked sialic acid receptors indicating the avian receptor specificity (Figure 2), showing that it possesses avian receptor specificity. We further evaluated the pathogenicity of B51 in a mouse model. The body weights of the virus-inoculated BALB/c mice decreased in 3dpi and then increased gradually. At 14 dpi, the body weights of the infected mice were comparable to those of the controls (Figure 3A). The positive control A/chicken/Heilongjiang/A147/2016(H9N2) replicated in the lung and nasal turbinate of infection mice at 3 dpi, but no virus was detected in the organs with the infection of B51 (Figure 3B). These results indicate that mice could not be infected by B51, and the virus showed low pathogenicity in BALB/c mice.

## Discussion

**FIGURE 4:**
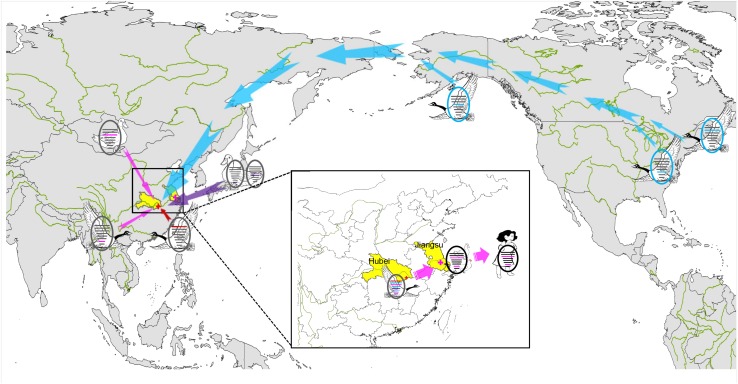
Sampling point and the proposed reassortment and transmission of H9N2 isolate among North America and Eurasia. Sampling point is marked with red cross, and the location of the first case of human infection with influenza A subtype H7N4 virus is marked with pink cross. Transmission routes for viral genes are indicated with arrow, North America-origin segments are colored in blue, Japan-origin segments are colored in purple, Jiangxi-origin segment is colored in red, and H7N4 related segments are colored in pink.

In December 2017, a novel reassortant influenza A(H9N2) virus was isolated from migratory waterfowl in an active AIV surveillance program in Hubei Province, China. Phylogenetic analysis showed that the individual gene segments of the virus had multiple origins, which differed by location, subtype and host species. It also indicated that the emergence of this reassortment H9N2 virus might have occurred in early 2016 according to tMRCA data. A novel reassortant H7N4 virus was identified from a 68 years-old woman and her backyard poultry (chickens and ducks) in Jiangsu Province, China, in January 2018. Importantly, the PA and M genes of the novel H9N2 isolate show the highest nucleotide similarity to those of the avian and human H7N4 strains. The MCC phylogenetic trees indicated that the two H7N4-like genes (PA and M genes) were most likely transferred from wild waterfowl to the poultry AIV gene pool in Jiangsu Province at the beginning of 2017, revealing the following sequence of spread for the virus segments: migratory birds to poultry to humans (Figure 4). Migratory birds play a key role in the spread of AIVs to different regions in the world, whereas the residential wild birds and domestic poultry influenza virus gene pool along the migratory routes also contributes to viral reassortment. Hubei Province, located in central China, has widely distributed lakes and wetlands providing an ideal habitat for migratory birds on the East Asia-Australasian flyway. Only one such H9N2 virus was isolated in our study suggesting that we need to strengthen AIV research and surveillance in wild birds in the Hubei Province and across the surrounding areas in order to detect novel reassortant influenza strains.

## Data Availability Statement

The datasets generated for this study can be found in the GISAID EpiFlu database (accession numbers: EPI1364378–EPI1364385).

## Ethics Statement

All animal studies were approved by the Institutional Animal Care and Use Committee of the Harbin Veterinary Research Institute, Chinese Academy of Agricultural Sciences. All animal procedures were carried out in strict accordance with the recommendations in the Guide for the Care and Use of Laboratory Animals of the Ministry of Science and Technology of the People’s Republic of China. All experiments were performed in a biosafety level 2+ laboratory (enhanced animal biosafety level 2 laboratory and a negative pressure-ventilation laboratory) at Harbin Veterinary Research Institute (Harbin, China).

## Author Contributions

XLi, JS, XLv, and YW performed the phylogenetic analyses and wrote the drafts of the manuscript. WL, XZ, YoL, GY, GC, JZ, and HZ collected the surveillance samples. MZ, XY, TF, PM, and YiL contributed on isolation and identification of virus. GZ, YH, and SY performed the phylogenetic analyses. YuL and ML performed the animal experiment. YaL, JR, and HC commented on and revised the drafts of the manuscript.

## Conflict of Interest

The authors declare that the research was conducted in the absence of any commercial or financial relationships that could be construed as a potential conflict of interest.

## References

[B1] Chai H. (2012). *Molecular Epidemiological Study on Influenza Virus in Wild Birds of Heilongjiang. doctor.* Harbin: Northeast Forestry University.

[B2] Chen H. Yuan H. Gao R. Zhang J. Wang D. Xiong Y. (2014). Clinical and epidemiological characteristics of a fatal case of avian influenza a H10N8 virus infection: a descriptive study. *Lancet* 383 714–721. 10.1016/S0140-6736(14)60111-2 24507376

[B3] Drummond A. J. Ho S. Y. W. Phillips M. J. Rambaut A. (2006). Relaxed phylogenetics and dating with confidence. *PLoS Biol.* 4:e88. 10.1371/journal.pbio.0040088 16683862 PMC1395354

[B4] Drummond A. J. Rambaut A. (2007). BEAST: bayesian evolutionary analysis by sampling trees. *BMC Evol. Biol.* 7:214. 10.1186/1471-2148-7-214 17996036 PMC2247476

[B5] Edgar R. C. (2004). MUSCLE: multiple sequence alignment with high accuracy and high throughput. *Nucleic Acids Res.* 32 1792–1797. 10.1093/nar/gkh340 15034147 PMC390337

[B6] Fan S. Deng G. Song J. Tian G. Suo Y. Jiang Y. (2009). Two amino acid residues in the matrix protein M1 contribute to the virulence difference of H5N1 avian influenza viruses in mice. *Virology* 384 28–32. 10.1016/j.virol.2008.11.044 19117585

[B7] Global Consortium for H5N8, and Related Influenza Viruses. (2016). Role for migratory wild birds in the global spread of avian influenza H5N8. *Science* 354 213–217. 10.1126/science.aaf8852 27738169 PMC5972003

[B8] Guan Y. Shortridge K. F. Krauss S. Webster R. G. (1999). Molecular characterization of H9N2 influenza viruses: were they the donors of the “internal” genes of H5N1 viruses in Hong Kong? *Proc. Natl Acad. Sci. U S A.* 96 9363–9367. 10.1073/pnas.96.16.9363 10430948 PMC17788

[B9] Homme P. J. Easterday B. C. (1970). Avian influenza virus infections. i. characteristics of influenza A-turkey-wisconsin-1966 virus. *Avian Dis.* 14 66–74. 10.2307/1588557 4314007

[B10] Jiao P. Tian G. Li Y. Deng G. Jiang Y. Liu C. (2008). A single-amino-acid substitution in the NS1 protein changes the pathogenicity of H5N1 avian influenza viruses in mice. *J. Virol.* 82 1146–1154. 10.1128/JVI.01698-1697 18032512 PMC2224464

[B11] Kingman J. F. C. (1982). The coalescent. *Stoch Process Their Appl.* 13 235–248. 10.1016/0304-4149(82)90011-90014

[B12] Kuo R. L. Krug R. M. (2009). Influenza a virus polymerase is an integral component of the F30-NS1A protein complex in infected cells. *J. Virol.* 83 1611–1616. 10.1128/JVI.01491-1498 19052083 PMC2643760

[B13] Lam T. T. Wang J. Shen Y. Zhou B. Duan L. Cheung C. L. (2013). The genesis and source of the H7N9 influenza viruses causing human infections in China. *Nature* 502 241–244. 10.1038/nature12515 23965623 PMC3801098

[B14] Li C. Wang S. Bing G. Carter R. A. Wang Z. Wang J. (2017). Genetic evolution of influenza H9N2 viruses isolated from various hosts in China from 1994 to 2013. *Emerg. Microbes Infect.* 6:e106. 10.1038/emi.2017.94 29184157 PMC5717095

[B15] Li X. Cui P. Zeng X. Jiang Y. Li Y. Yang J. (2019). Characterization of avian influenza H5N3 reassortants isolated from migratory waterfowl and domestic ducks in China from 2015 to 2018. *Transbound. Emerg. Dis.* 66 2605–2610. 10.1111/tbed.13324 31402584

[B16] Li X. Shi J. Guo J. Deng G. Zhang Q. Wang J. (2014). Genetics, receptor binding property, and transmissibility in mammals of naturally isolated H9N2 avian influenza viruses. *PLoS Pathog.* 10:e1004508. 10.1371/journal.ppat.1004508 25411973 PMC4239090

[B17] Li Z. Chen H. Jiao P. Deng G. Tian G. Li Y. (2005). Molecular basis of replication of duck H5N1 influenza viruses in a mammalian mouse model. *J. Virol.* 79 12058–12064. 10.1128/JVI.79.18.12058-12064.2005 16140781 PMC1212590

[B18] Liu J. Xiao H. Lei F. Zhu Q. Qin K. Zhang X. W. (2005). Highly pathogenic H5N1 influenza virus infection in migratory birds. *Science* 309:1206. 10.1126/science.1115273 16000410

[B19] Ramey A. M. Reeves A. B. Donnelly T. Poulson R. L. Stallknecht D. E. (2018). Introduction of eurasian-origin influenza a(H8N4) virus into North America by migratory birds. *Emerg. Infect. Dis.* 24 1950–1953. 10.3201/eid2410.180447 30226185 PMC6154152

[B20] Shapiro B. Rambaut A. Drummond A. J. (2006). Choosing appropriate substitution models for the phylogenetic analysis of protein-coding sequences. *Mol. Biol. Evol.* 23 7–9. 10.1093/molbev/msj021 16177232

[B21] Shen Y. Y. Ke C. W. Li Q. Yuan R. Y. Xiang D. Jia W. X. (2016). Novel reassortant avian influenza A(H5N6) viruses in humans, guangdong, China, 2015. *Emerg. Infect. Dis.* 22 1507–1509. 10.3201/eid2208.160146 27331418 PMC4982152

[B22] Stamatakis A. (2014). RAxML version 8: a tool for phylogenetic analysis and post-analysis of large phylogenies. *Bioinformatics* 30 1312–1313. 10.1093/bioinformatics/btu033 24451623 PMC3998144

[B23] Subbarao E. K. London W. Murphy B. R. (1993). A single amino acid in the PB2 gene of influenza a virus is a determinant of host range. *J. Virol.* 67 1761–1764. 8445709 10.1128/jvi.67.4.1761-1764.1993 PMC240216

[B24] Vines A. Wells K. Matrosovich M. Castrucci M. R. Ito T. Kawaoka Y. (1998). The role of influenza a virus hemagglutinin residues 226 and 228 in receptor specificity and host range restriction. *J. Virol.* 72 7626–7631. 9696865 10.1128/jvi.72.9.7626-7631.1998 PMC110023

[B25] World Health Organization [WHO] (2002). *WHO Animal Influenza Manual* Available at: http://www.who.int/csr/resources/publications/influenza/en/whocdscsrncs20025rev.pdf

[B26] Yamada S. Hatta M. Staker B. L. Watanabe S. Imai M. Shinya K. (2010). Biological and structural characterization of a host-adapting amino acid in influenza virus. *PLoS Pathog.* 6:e1001034. 10.1371/journal.ppat.1001034 20700447 PMC2916879

