# Supplementary material for: Novel Reassortant Avian Influenza A(H9N2) Virus Isolate in Migratory Waterfowl in Hubei Province, China
Source: Front Microbiol. 2020 Feb 13;11:220. doi: 10.3389/fmicb.2020.00220 (PMC7031422; doi:10.3389/fmicb.2020.00220)
Supplement: Supplementary file 1 [file Table_1.docx]

Supplementary Material

Supplementary Figure 1. Unrooted maximum-likelihood phylogenetic trees with colourful branch symbols as tip labels showing inferred relationship among nucleotide sequences for the complete coding regions of the gene segments for influenza A virus strain A/wild waterfowl/Hubei/B51/2017(H9N2) (shown in red branch). Reference sequences from viruses isolated from wild birds in Eurasia (blue star) or Americas (pink star), and poultry in Eurasia (blue circle) or Americas (red circle). Bootstrap support values ≥70 are shown. Segments shown are A) polymerase basic (PB2); B) polymerase basic (PB1); C) polymerase (PA); D) hemagglutinin (HA); E) nucleoprotein (NP); F) neuraminidase (NA); G) matrix protein (M); H) nonstructural protein (NS).

Supplementary Figure 2. Maximum clade credibility trees of the coding sequences of 8 segments. Node bars indicate 95% highest posterior density (HPD) of the node height. Influenza A virus strain A/wild waterfowl/Hubei/B51/2017(H9N2) was coloured in red. Each branch was coloured by posterior probability. Segments shown are A) polymerase basic (PB2); B) polymerase basic (PB1); C) polymerase (PA); D) hemagglutinin (HA); E) nucleoprotein (NP); F) neuraminidase (NA); G) matrix protein (M); H) nonstructural protein (NS).

Supplementary Figure 3. Map of sampling sites in Hubei Province. A) Paihu Wetland; B) Honghu Weiland; C) Shangshehu Wetland; D) Fufu Wetland; E) Liangzihu Wetland; F) Wanghu Wetland; G) Longganhu Wetland.

Supplementary Table 1: Surveillance of avian influenza virus in wild waterfowl in Hubei Province, China

| Collection date | Collection site | Longitude, latitude | Type of simple* | Samples | Positive samples  (Isolation rate) | Subtype |
| --- | --- | --- | --- | --- | --- | --- |
| Dec, 2017 | Liangzihu Wetland | 114.61E, 30.14N | O&C | 139 | 0 | - |
|  | Wanghu Wetland | 115.21E, 29.83N | FD | 138 | 6 (4.348%) | H1N3 (5), H9N2 (1) |
|  |  |  | O&C | 135 | 0 | - |
| Jan, 2018 | Paihu Wetland | 113.45E, 30.37N | FD | 41 | 0 | - |
|  | Honghu Wetland | 113.45E, 29.81N | FD | 256 | 0 | - |
|  | Fuhe Wetland | 114.69E, 30.32N | FD | 149 | 1 (0.671%) | H4N6 (1) |
|  | Wanghu Wetland | 115.21E, 29.83N | FD | 181 | 0 | - |
|  | Shangshehu Wetlang | 114.31E, 30.35N | FD | 2 | 0 | - |
| Feb, 2018 | Wanghu Wetland | 115.21E, 29.83N | FD | 1138 | 0 | - |
|  | Liangzihu Wetland | 114.61E, 30.14N | O&C | 87 | 0 | - |
| Mar, 2018 | Wanghu Wetland | 115.21E, 29.83N | O&C | 8 | 0 | - |
|  | Liangzihu Wetland | 114.61E, 30.14N | O&C | 22 | 0 | - |
|  | Longganhu Wetland | 115.90E, 29.89N | O&C | 2 | 0 | - |
| Total | | | | 2298 | 7 (0.305%) |  |
| *: FD, fresh dropping; O&C, mixed oropharyngeal and cloacal swab for a single host | | | | | | |

# Supplementary Table 2: Amino acid anaysis of influenza A virus strain A/wild waterfowl/Hubei/B51/2017(H9N2)

| **Protein** | **Amino acid position/motif** | **Phenotypic consequences** | **Isolate** |
| --- | --- | --- | --- |
| PB2 | D256G | Enhanced polymerase activity, mammalian host adaptation | D |
|  | Q591K | Enhanced replication efficiency, increased virulence in mice | Q |
|  | E627K | Mammalian host adaptation, increased virulence in mice | E |
|  | D701N | Mammalian host adaptation, increased virulence in mice | D |
| PB1-F2 | N66S | Increased virulence and antivirus response in mice | N |
| HA | Cleavage site | Polybasic cleavage motif sequence required for high pathogenicity of avian influenza viruses | PAASDR |
|  | Q226L (H3 numbering) | Increased virus binding to α2-6 | Q |
|  | G228S (H3 numbering) | Increased virus binding to α2-6 | G |
| NA | Neck deletion | Enhanced virulence in mice | No deletion |
|  | V116A (N2 numbering) | Reduced susceptibility to zanamivir and oseltamivir | V |
|  | E119A/G/V (N2 numbering) | Reduced susceptibility to zanamivir, oseltamivir and/or peramivir | E |
|  | Q136L/K/R (N2 numbering) | Reduced susceptibility to zanamivir and oseltamivir | Q |
|  | R156K (N2 numbering) | Reduced susceptiblity to oseltamivir, zanamivir, and peramivir | R |
|  | I222M/V/L/K/R (N2 numbering) | Reduced susceptibility to oseltamivir | I |
|  | H274Y/R (N2 numbering) | Reduced susceptibility to oseltamivir and peramivir | H |
|  | E277Q (N2 numbering) | Reduced susceptibility to oseltamivir | E |
| M1 | **N30D** | **Increased virulence in mice** | **D** |
|  | T139A | Increased virulence in mice | T |
|  | **T215A** | **Increased virulence in mice** | **A** |
| M2 | L26F | Reduced susceptibility to amantadine and rimantadine | L |
|  | V27A | Reduced susceptibility to amantadine and rimantadine | V |
|  | A30V/T/S | Reduced susceptibility to amantadine and rimantadine | A |
|  | S31N/G | Reduced susceptibility to amantadine and rimantadine | S |
|  | G34E | Reduced susceptibility to amantadine and rimantadine | G |
| NS1 | 80-84 deletion | Increased virulence in mice | No deletion |
|  | **P42S** | **Increased virulence in mice** | **S** |
|  | D87E | Increased virulence in mice | D |
|  | **L98F** | **Increased virulence in mice** | **F** |
|  | **I101M** | **Increased virulence in mice** | **M** |
