# Supplementary figures and images for: Novel Reassortant Avian Influenza A(H9N2) Virus Isolate in Migratory Waterfowl in Hubei Province, China
Source: Front Microbiol. 2020 Feb 13;11:220. doi: 10.3389/fmicb.2020.00220 (PMC7031422; doi:10.3389/fmicb.2020.00220)

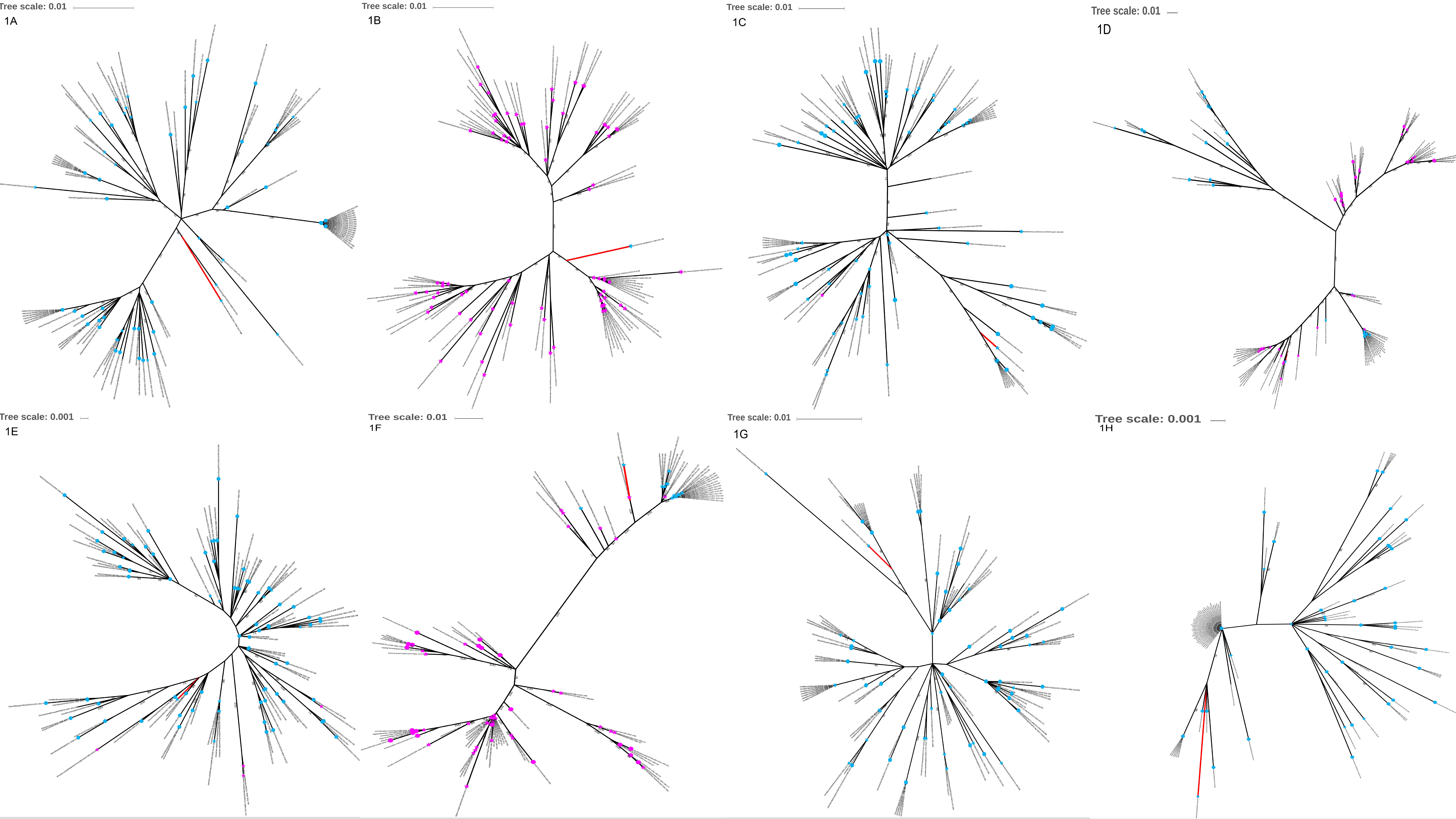

Supplement: Supplementary file 2 [file Image_1.TIF]

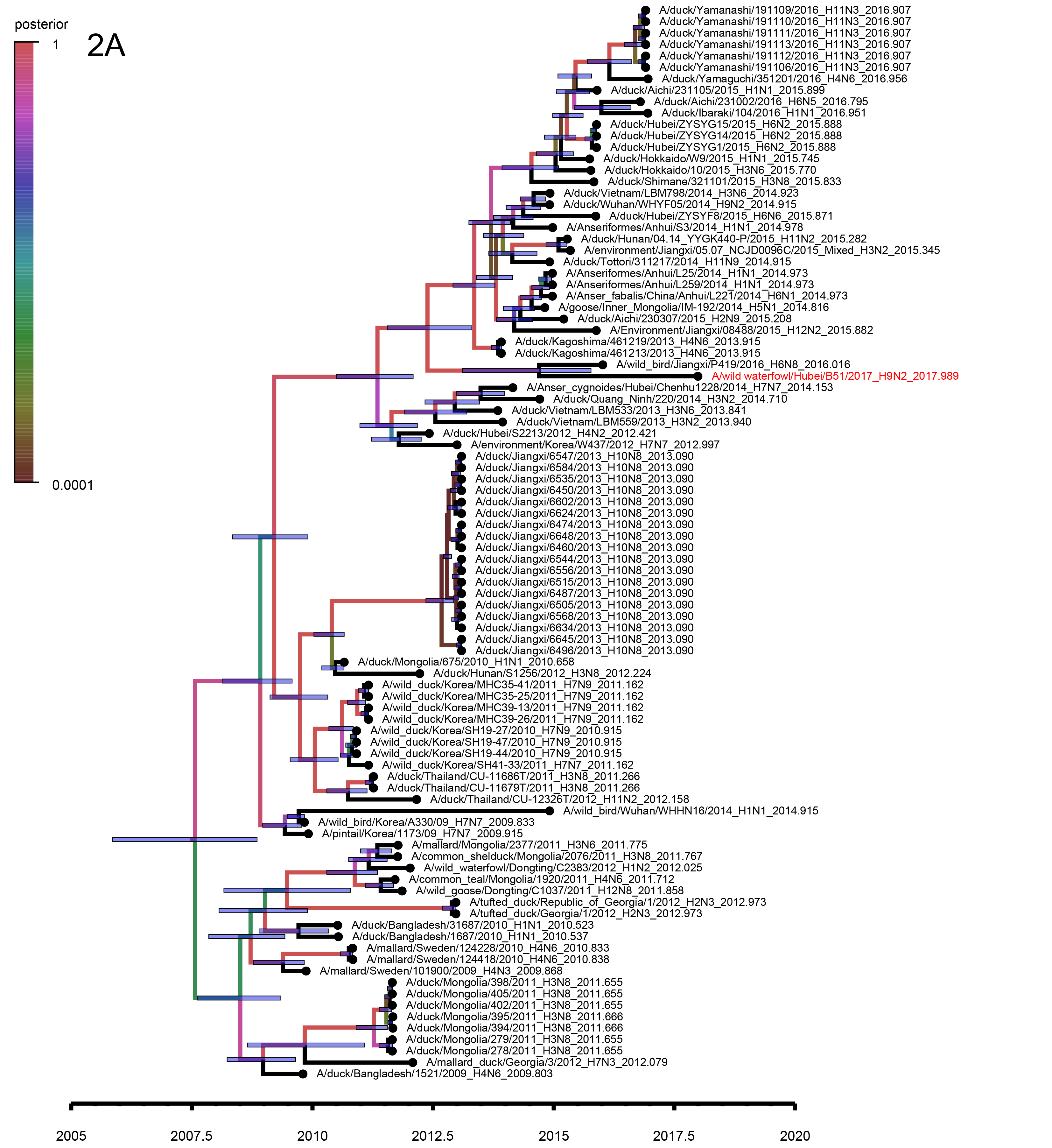

Supplement: Supplementary file 3 [file Image_2.TIF]

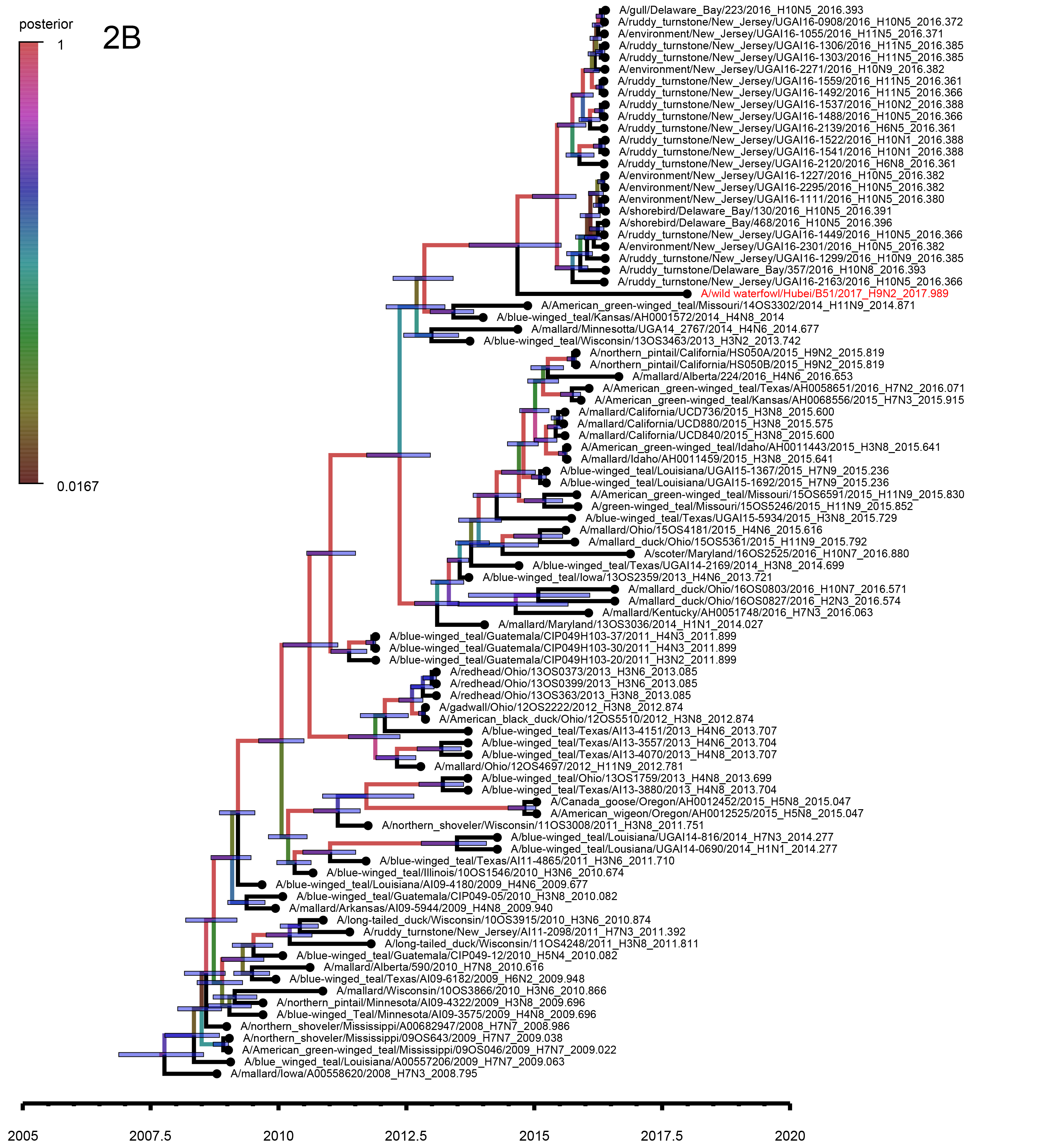

Supplement: Supplementary file 4 [file Image_3.TIF]

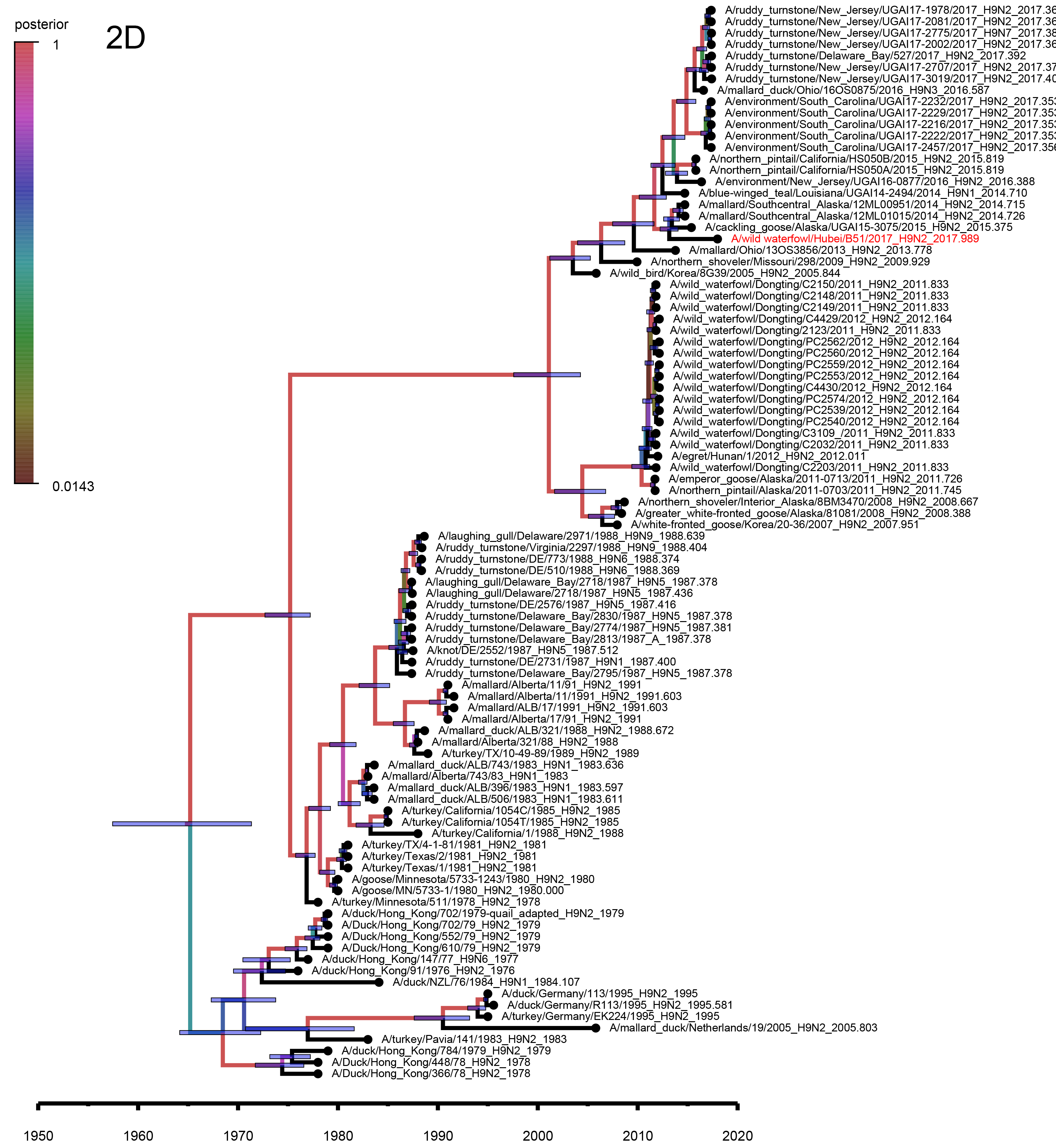

Supplement: Supplementary file 5 [file Image_5.TIF]

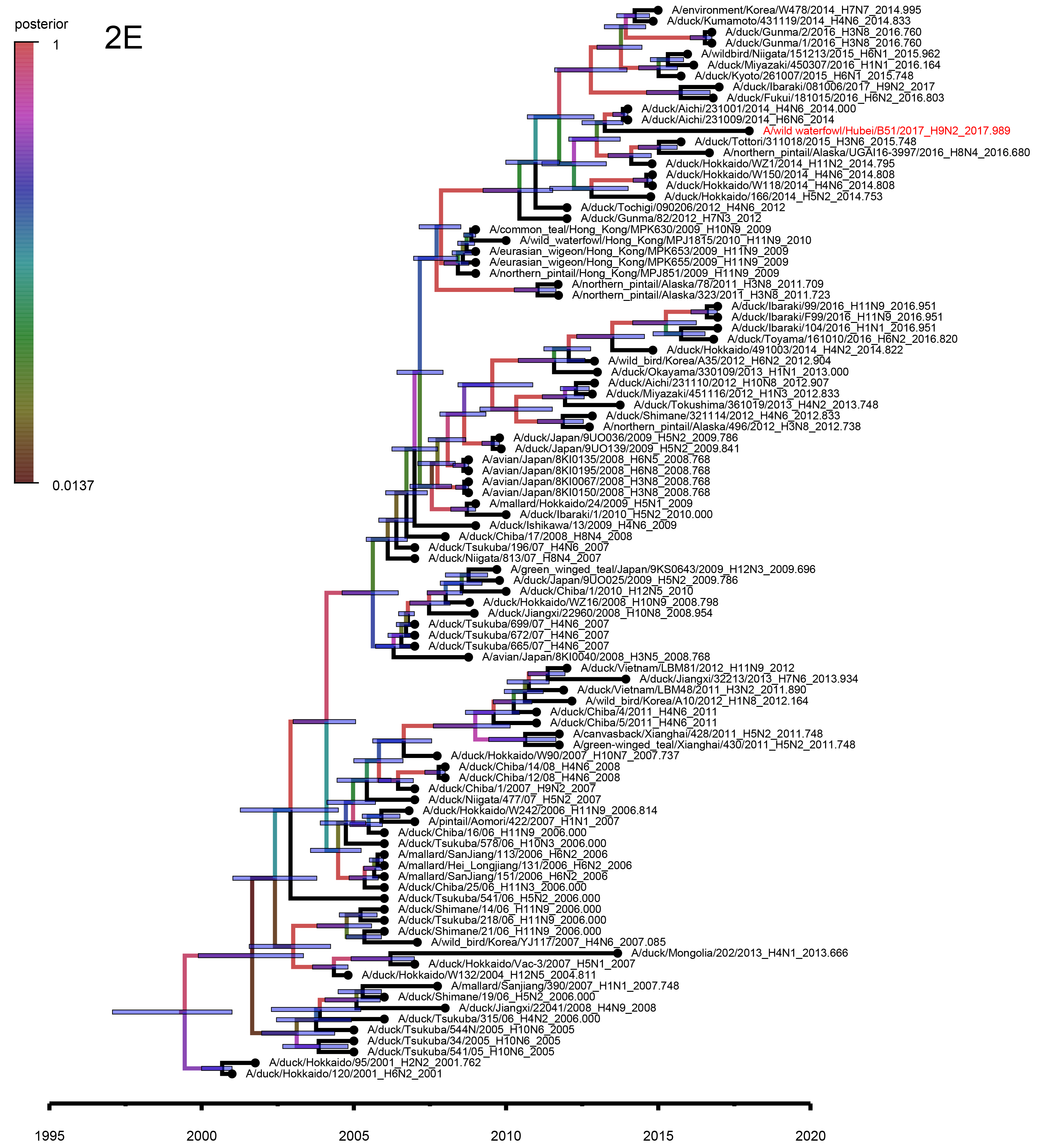

Supplement: Supplementary file 6 [file Image_6.TIF]

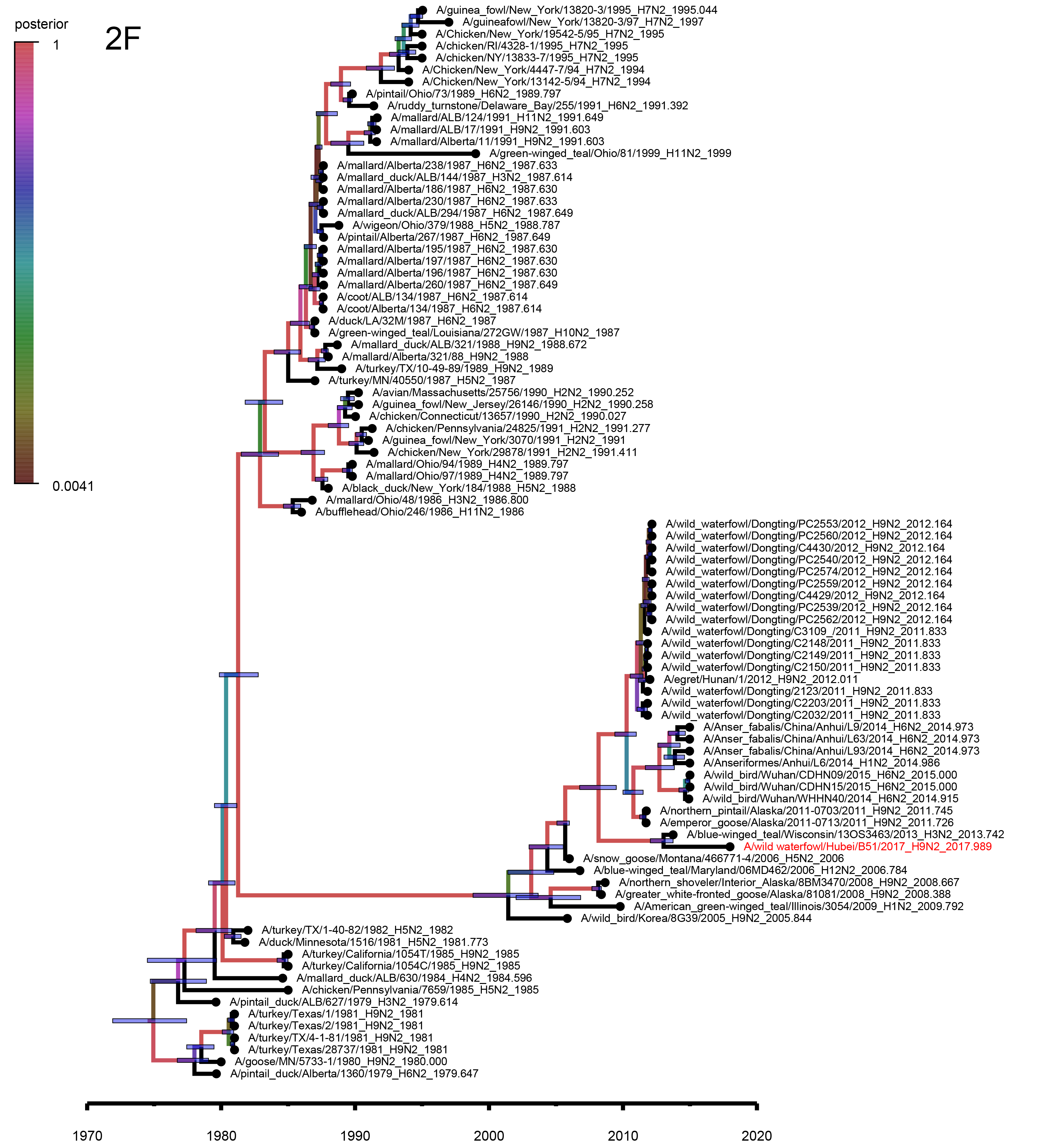

Supplement: Supplementary file 7 [file Image_7.TIF]

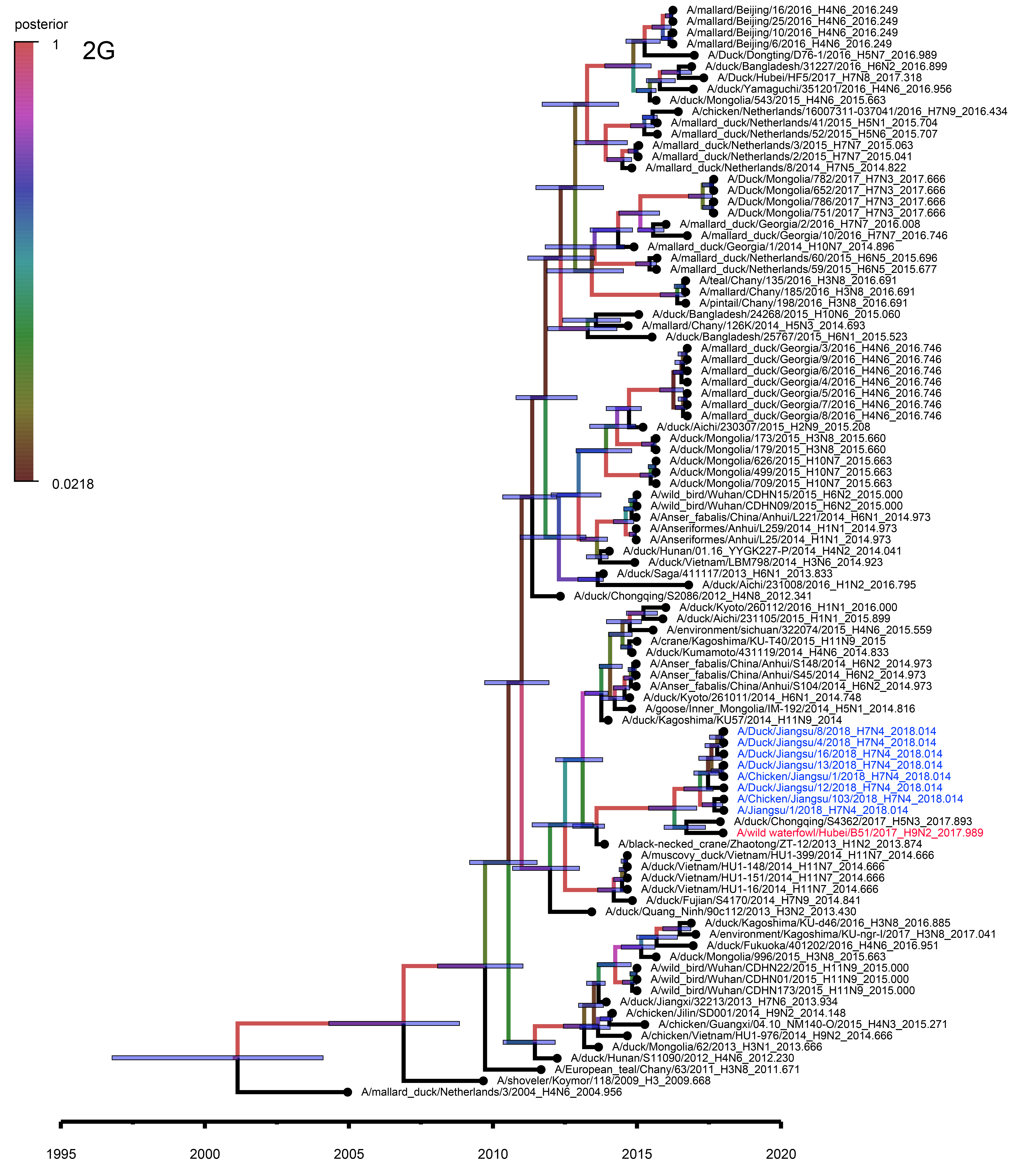

Supplement: Supplementary file 8 [file Image_8.TIF]

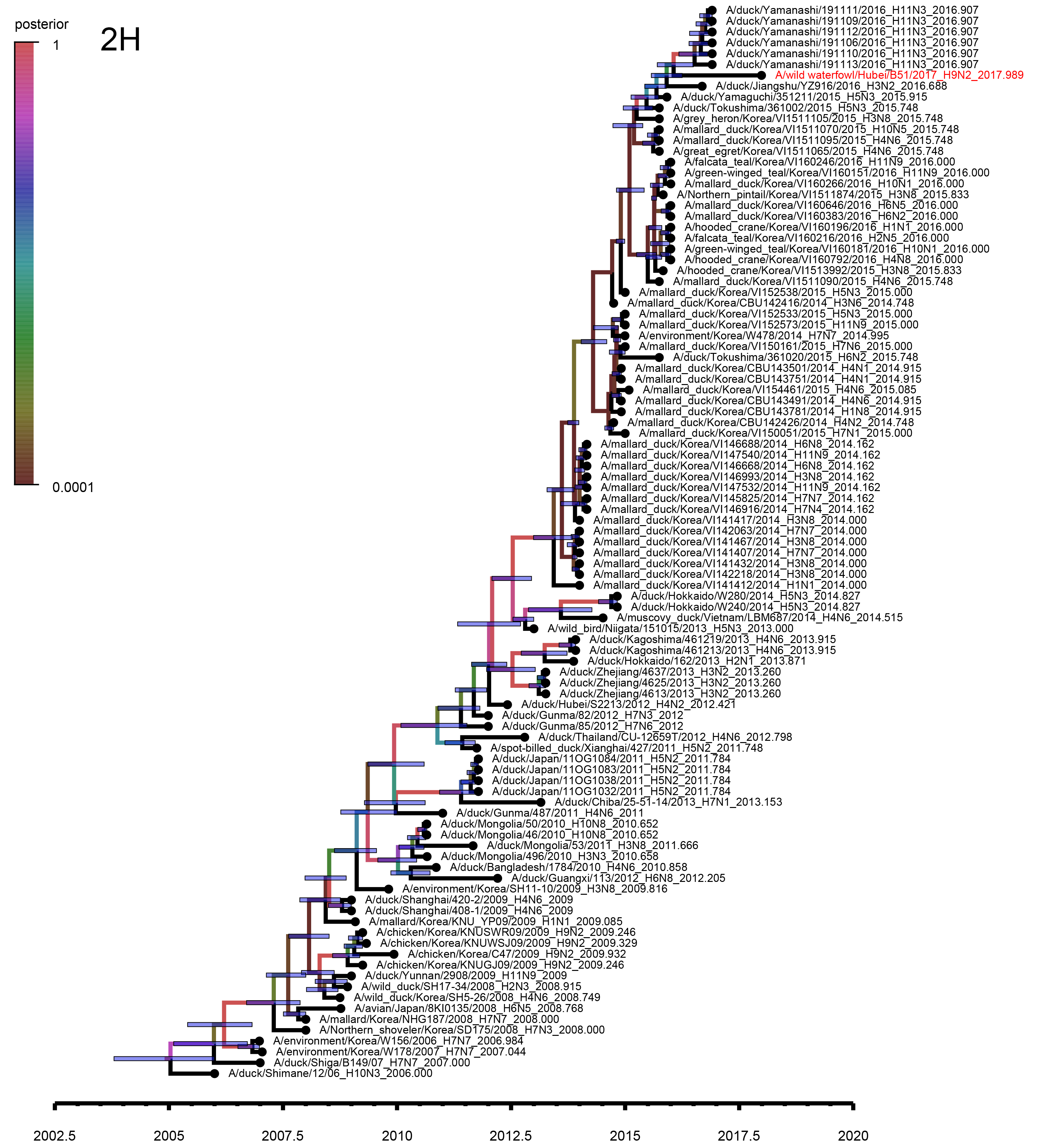

Supplement: Supplementary file 9 [file Image_9.TIF]

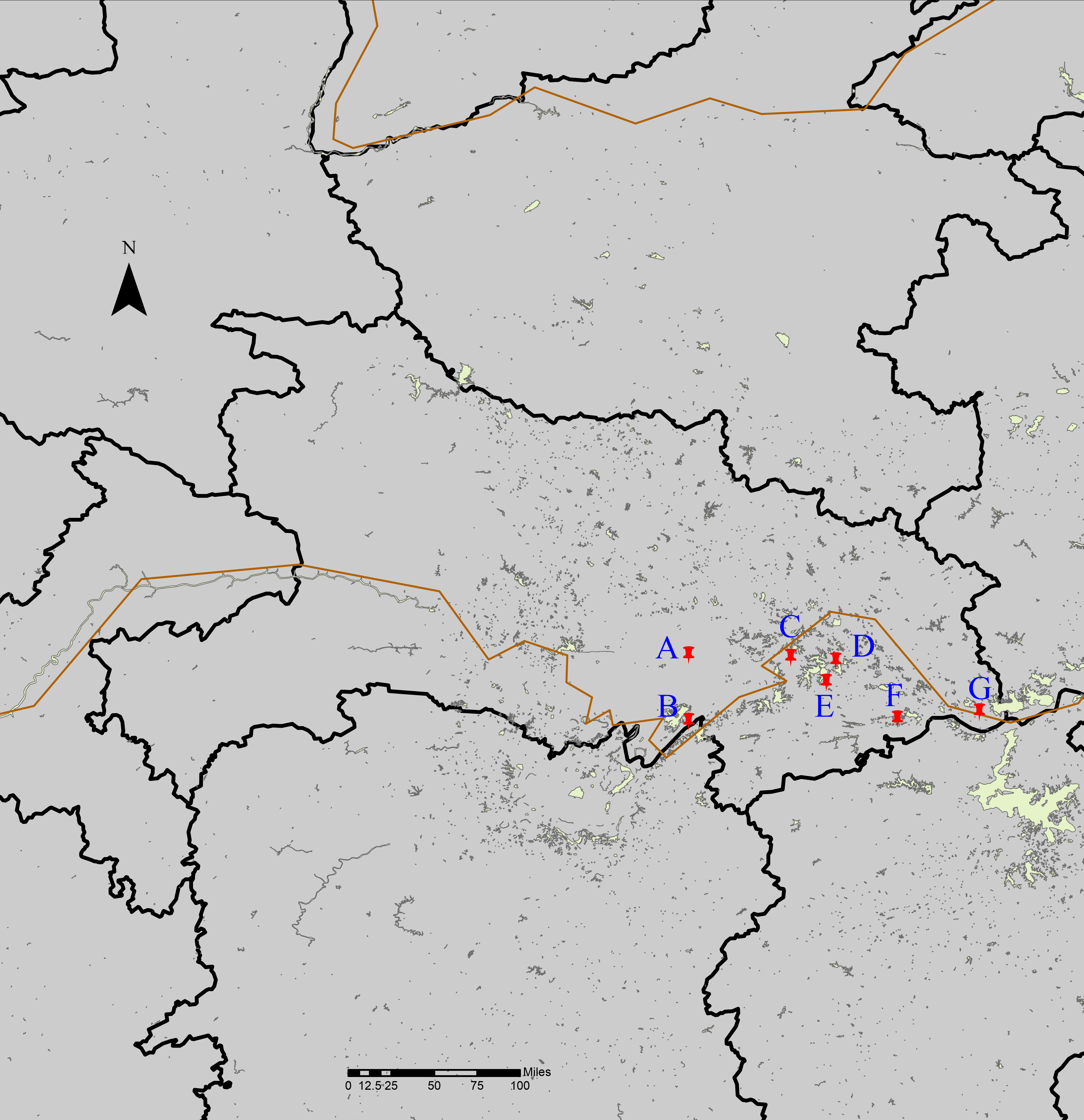

Supplement: Supplementary file 10 [file Image_10.TIFF]
